# High Lifetime Prevalence of Syphilis in Men Who Have Sex With Men and Transgender Women Versus Low Lifetime Prevalence in Female Sex Workers in Lima, Peru

**DOI:** 10.1097/OLQ.0000000000001200

**Published:** 2020-06-15

**Authors:** Phoebe Hung, Ethan Osias, Kelika A. Konda, Gino M. Calvo, E. Michael Reyes-Díaz, Silver K. Vargas, Cameron Goldbeck, Carlos F. Caceres, Jeffrey D. Klausner

**Affiliations:** From the ∗ Division of Infectious Diseases, David Geffen School of Medicine, University of California Los Angeles, CA; † Center for Interdisciplinary Investigation in Health, Sexuality, and AIDS, Universidad Peruana Cayetano Heredia, Lima, Peru

## Abstract

The syphilis epidemic continues to cause substantial morbidity worldwide and is worsening despite ongoing control efforts. Syphilis remains an important public health problem among 3 key populations: men who have sex with men (MSM), transgender women, and female sex workers.

**Methods:**

We conducted a retrospective chart review of patients that received rapid point-of-care treponemal antibody tests from January 2019 to July 2019 in 4 sexually transmitted infection (STI) clinics in Lima, Peru. We assessed patient medical records for human immunodeficiency virus (HIV) infection, history of STIs, as well as sociodemographic and behavioral characteristics. Cross-sectional descriptive analyses were used to determine factors associated with treponemal positivity.

**Results:**

We included 401 patient records in our analyses: 252 MSM, 31 transgender women, and 118 female sex workers. The overall median age of patients was 29.0 years (interquartile range, 24.0–36.0 years). Positivity on the treponemal test was 28.9% (95% confidence interval [CI], 24.3%–33.3%) overall, 37.7% (95% CI, 31.7%–44.0%) for MSM, 54.8% (95% CI, 36.0%–72.7%) for transgender women, and 3.4% (95% CI, 0.9%–8.5%) for female sex workers. In the bivariate analysis, treponemal positivity was also associated with receptive anal sex in the last 6 months in MSM (*P* < 0.01). Additionally, treponemal positivity increased with age (*P* = 0.0212) and varied by socioeconomic status (*P* < 0.01). Multivariate Least Absolute Shrinkage and Selection Operator logistic regression showed that treponemal positivity was highly associated with HIV coinfection (adjusted odds ratio, 5.42) and previous STI other than HIV or syphilis (adjusted odds ratio, 1.54).

**Conclusions:**

A review of the medical records of members of 3 key populations who had recently received a rapid point-of-care treponemal test in Lima, Peru, revealed that lifetime prevalence of syphilis was high among MSM and transgender women, but low among female sex workers. Those results may indicate a need for more frequent, regular testing among MSM and transgender women—possibly in conjunction with HIV testing, and appropriate treatment of those shown to be positive.

## Abstract 2

A study of men who have sex with men, transwomen, and female sex workers in Lima, Peru found that lifetime prevalence of syphilis was substantially lower in female sex workers compared with other groups.

Syphilis remains a serious cause of morbidity worldwide^1^ with a global annual incidence of 5.6 million new cases per year^2^ and total prevalence of 56.1 million.^3^ The most affected are often members of 3 key populations: men who have sex with men (MSM), transwomen, and female sex workers.^4,5^ A recent study done in Lima, Peru reported a baseline prevalence of recently acquired syphilis of 16.8% for MSM, and 6.7% for transgender women.^6^ Factors, such as condomless anal sex, human immunodeficiency virus (HIV) infection, previous sexually transmitted infections (STIs), and lower education level, have also been associated with increased syphilis.^6–8^

Syphilis is a bacterial infection caused by *Treponema pallidum,* subsp. *pallidum* (*T. pallidum*). Current diagnostic protocols include a rapid point-of-care treponemal antibody test that identifies host antibody response to *T. pallidum* antigens,^9^ which indicates a lifetime history of syphilis. A meta-analysis conducted in 2013 showed the rapid treponemal antibody test to be of comparable sensitivity (90.04%) and specificity (94.15%) to those of laboratory based treponemal tests such as the *T. pallidum* particle agglutination assay and *T. pallidum* hemagglutination assay.^10^

In this study, we described the lifetime prevalence of syphilis based on positivity on a rapid point-of-care treponemal test and factors associated with positivity. Our sample came from 3 key populations: MSM, transgender women, and female sex workers. Characterization of those populations could help improve prevention and control measures and help to stem the growth of the syphilis epidemic. Here, we report baseline characteristics and behaviors associated with positivity on the rapid treponemal antibody test based on chart review of individuals who received a rapid treponemal antibody test in 1 of 4 STI clinics that treat predominantly lower-income MSM, transwomen, and female sex workers in Lima, Peru.

## METHODS AND DESIGN

### Study Procedure

We conducted a retrospective chart review at 4 STI clinics in Lima, Peru that are easily accessible to local residents. Three of those clinics—Tahuantinsuyo Health Center, Alberto Barton Health Center, and Caja de Agua Health Center—are government health centers located in low-income neighborhoods in Lima and serve MSM, transwomen and female sex workers. The fourth clinic, Epicentro, is a community-based, nongovernmental health center located in the middle-income district of Barranco that provides fee-based services to MSM and transwomen. All provide regular clinical check-up appointments for HIV and other STI screening and counseling, as well as clinical STI treatment and referral for HIV treatment. All centers provide preexposure prophylaxis (PrEP) or PrEP referral in the context of research or demonstration studies as PrEP is not available from the Peruvian Ministry of Health.

### Study Population

As part of a larger study, PICASSO 2 (NIH award number: 5R01AI139265-02), we reviewed the records of patients that received a rapid point-of-care treponemal test (Determine Syphilis TP, Abbott, US) in Lima, Peru from January 2019 to July 2019. All rapid treponemal tests were performed by trained laboratory staff at each of the 4 clinics. Patients with positive rapid treponemal tests received a confirmatory rapid plasma reagin (RPR) test (RPR Quicktest, Stanbio, US). The laboratory staff diluted the specimen to the lowest positive titer and reported the results on the patient chart. The attending physicians designated the classification of syphilis based on the rapid treponemal test results, RPR results, and clinical presentation. Clinicians diagnosed patients as “serofast” when patients had a positive RPR titer but had been recently diagnosed with an active infection and received appropriate treatment. Clinicians considered patients with a positive treponemal rapid test and a nonreactive RPR titer to not have an active syphilis infection and were not diagnosed with syphilis. These patients, however, were considered to have an active syphilis infection in the past.

Eligible patient medical records came from MSM, transwomen, or female sex workers 18 years or older. Because we looked at records that came from those specific populations, patient medical records were excluded if they were a heterosexual man with only female sex partners or a heterosexual woman with no history of sex work. These were the clinical records that were accessible to the study team but do not represent all of the patients seen in this period. However, this is a random selection of clinical histories. If the patient had multiple visits in the given period, we used the information from their most recent visit.

Behavioral characteristics were based on information in the medical record. Trained professionals at each sexual health clinic conduct patient interviews during each patient's visit and record self-reported behavior from the last 3 months. All individuals at the 4 sexual health clinics were tested for syphilis and HIV regardless of reported behavior. The HIV coinfection status was based on record of positive HIV rapid test as reported in the clinical chart.

### Data Collection and Analyses

We reviewed medical records for demographic, STI history and treatment, past syphilis diagnoses and treatment, and sexual behavior data. Patient demographic data included age, gender, education level, socioeconomic status based on district of residence, and medical insurance. Sexually transmitted infection history included past diagnoses of syphilis, HIV infection and antiretroviral therapy treatment history, chlamydia, gonorrhea, hepatitis B, genital herpes, and human papilloma virus infection. Sexual behavior data included primary role during anal sex (ie, insertive, receptive, or both), history of sex work, and frequency of condom use in the last 3 months. We used average income levels of the district in which they lived to designate socioeconomic status.^11^

We abstracted medical records in a standardized fashion and entered all data through REDCap (https://www.project-redcap.org/), a web-based data management system that is currently used by both Universidad Peruana Cayetano Heredia and University of California, Los Angeles. REDCap supports the entry of clinical and laboratory data in a Health Insurance Portability and Accountability Act-compliant environment. Data are accessed through a password-protected web portal, making it secure and easy to share data between institutions in different countries.

### Statistical Analyses

Our primary objective of this study was to describe lifetime prevalence of syphilis in 3 key populations in Peru through prevalence of treponemal positivity and to highlight factors related to positivity. Our secondary objective was to compare the diagnosis and treatment of active and serofast syphilis with recommended guidelines in Peru.

For the descriptive analyses, we utilized χ^2^ tests. We analyzed the possible trend in increasing age and lifetime syphilis positivity using the Cochran-Armitage trend test. We dealt with missing data on a variable-by-variable basis. We included a group of “unknown data” for variables with more than 10% missing data (education, insurance, role during anal sex, condom usage). In variables with less than 10% missing data, we dropped missing cases from analysis. Given the nature of the clinical charts reviewed, missing data are assumed to be missing completely at random. Therefore, there is likely no bias in the missing results and no influence on the outcome of our analyses, except in decreasing the sample size.

Additionally, we conducted exploratory analyses using a Least Absolute Shrinkage and Selection Operator (LASSO) logistic regression model to find predictors of *T. pallidum* positivity status in MSM and transwomen.^12^ The LASSO regression adds a penalty hyperparameter to model parameters when performing estimation. Also known as a “shrinkage parameter,” its inclusion shrinks weak predictors to zero leaving few variables in the final than originally entered, thereby performing variable selection for strong predictors.^13^ We chose to construct a LASSO model as opposed to a standard multivariate regression because of LASSO's variable selection effect. Given our small sample size and few potential exposures of interest, standard regression models were limited in their complexity. In addition, we did not have strong hypotheses to test a single predictor of interest regarding multivariate analyses. Therefore, we chose a LASSO model to explore the relationships between these variables and for its variable selection effect. Female sex workers were excluded due to the statistically distinct nature of this population due to too few treponemal-positive patients leading to insufficient power. Population type, MSM and transwomen, was a particular predictor of interest to include in the model. To control for confounding by population type, we additionally included other candidate variables if their bivariate association with population type had a *P* value less than 0.05 was found or from an a priori hypothesis. Variables with a statistically significant association with population type are: education level, medical insurance, neighborhood income level, condom usage in the past 3 months and insertive or receptive role in anal sex. Variables included due to an *a priori* hypothesis are: history of an STI, population type, and HIV status.

The model selected the following variables: history of an STI, population type, education level, neighborhood income level, condom use in the past 3 months, insertive or receptive role in sex, and HIV status. The LASSO regression was used to determine adjusted odds ratios of the selected variables. As is common in LASSO models, confidence intervals (CIs) and significant values cannot be readily or meaningfully calculated and thus are not reported.

### Ethical Considerations

We obtained approval from the institutional review boards at University of California, Los Angeles, Universidad Peruana Cayetano Heredia, and Alberto Barton Health Center for the PICASSO 2 study (approval numbers: CIEI 103093, IRB 18-001225).

## RESULTS

We included 401 patient records in our analyses: 252 from MSM, 31 from transwomen, and 118 from female sex workers. The overall median age of patients was 29.0 years old (interquartile range: 24.0, 36.0). The median age for MSM was 29.0 (interquartile range: 25.0, 36.0), for transgender women 26.0 (interquartile range: 25.0, 34.0), and for female sex workers 28.0 (interquartile range: 23.0, 35.0). Concerning the STI clinics of origin, 38.2% of records belonged to Epicentro, 15.2% to Caja de Agua Health Center, 14.5% to Tahuantinsuyo Health Center, and 32.2% to Alberto Barton Health Center. In terms of socioeconomic status, 71.1% lived in lower income districts, 4.7% in middle income districts, and 16.0% in higher income districts, while the remaining 8.2% could not be determined.

**Figure 1:**
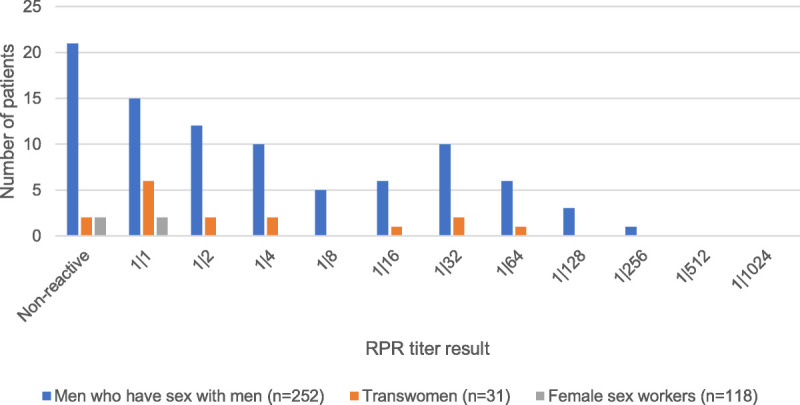
Number of patients with individual RPR results, if positive for treponemal antibody, Lima, Peru, January 2019 to July 2019.

Positivity on the treponemal antibody test was 28.9% (n = 116; 95% CI, 24.5%–33.6%) overall; and 37.7% (n = 95; 95% CI, 31.7%–44.0%) among MSM, 54.8% (n = 17; 95% CI, 36.0%–72.7%) among transwomen, and 3.4% (n = 4, 95% CI: 0.9%, 8.5%) among female sex workers. The distribution of RPR titers is shown in Figure 1. In terms of current syphilis diagnosis, 78.6% were not currently infected with syphilis, 10.1% were diagnosed as serofast, 0.5% with primary syphilis infection, 0.5% with secondary syphilis infection, 6.9% with latent syphilis infection, and no patients with tertiary syphilis or neurosyphilis. The majority of individuals do not have active syphilis. Of those diagnosed with active infection, most were diagnosed with latent syphilis, indicating the inability to accurately place the time of infection.

In terms of history of prior STI, 7.5% of all patients had a prior diagnosis of syphilis, 13.0% had been diagnosed with HIV infection, and 12.7% had a diagnosis of another STI other than HIV or syphilis in the past.

### Characteristics Associated With Treponemal Positivity

**Figure 2:**
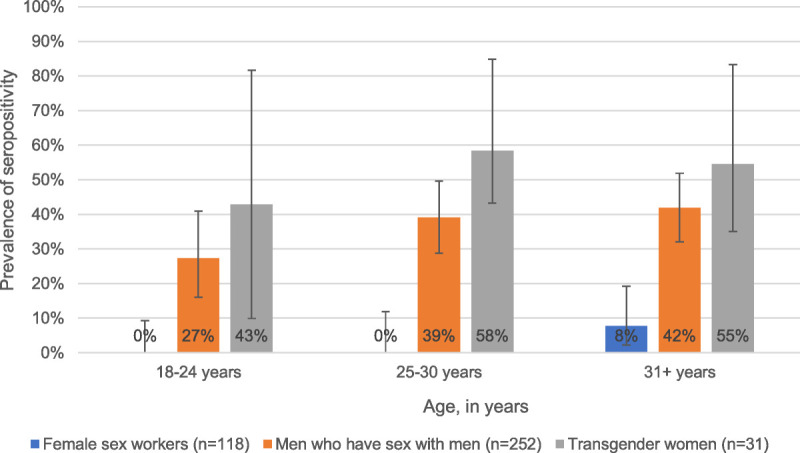
Prevalence of seropositivity with 95% CI on the *T. pallidum* antibody test among different age groups in female sex workers, MSM, and transgender women in Lima, Peru, January 2019 to July 2019.

Older age groups had higher levels of treponemal positivity overall (*P* = 0.019, Cochran-Armitage Trend Test). Among MSM, those who were older than 30 years had a frequency of treponemal positivity of 41.5% as opposed to a frequency of 27.3% in those 18 to 24 years (*P* = 0.086). This difference is not significant, but the data may be underpowered to show significance. In transwomen, those older than 30 years had a frequency of positivity of 54.5% compared with 42.9% in those 18 to 24 years. We observed no correlation between age and treponemal positivity in transgender women (*P* = 0.678), though because of a small sample, this test could be underpowered. Finally, 8.0% of older female sex workers were positive for treponemal antibodies compared with 0.0% in younger groups (*P* = 0.035; see Fig. 2).

Additionally, frequency of treponemal positivity varied by sexual behavior in MSM. Those who reported having exclusively insertive anal sex had a frequency of treponemal positivity of 26.9% as opposed to a frequency of 46.1% in those who reported either exclusive receptive anal sex or both receptive and insertive anal sex (*P* < 0.01). There was less variation in role in anal sex among transgender women. See Table 1 for frequencies of treponemal positivity of other socioeconomic characteristics.

**TABLE 1:**
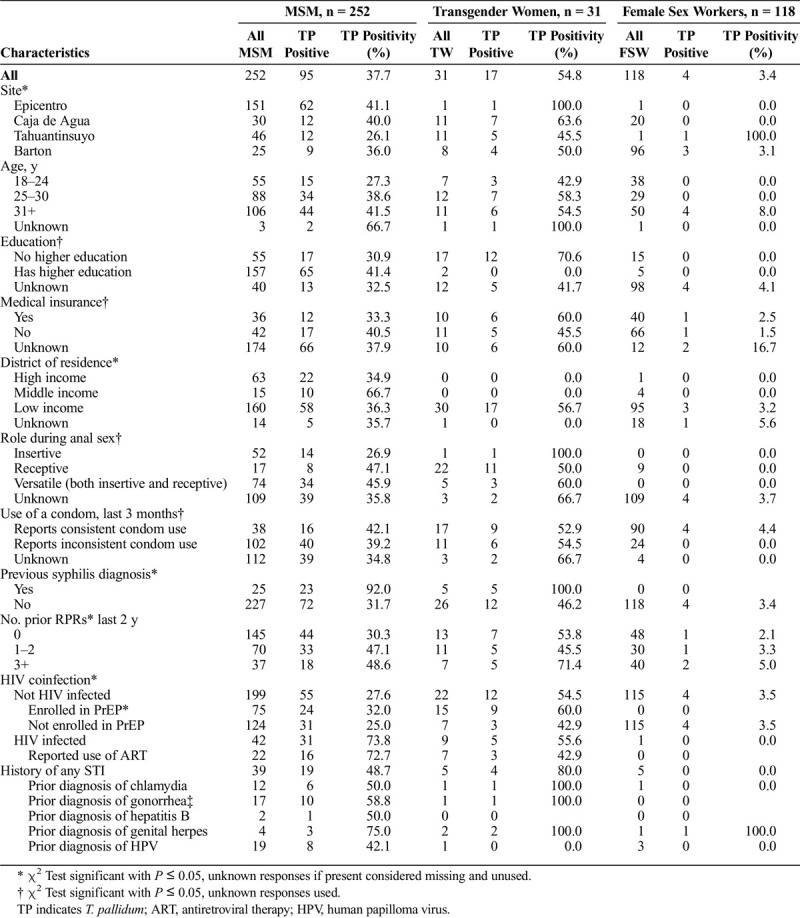
Frequency of *T. pallidum* Antibody Positivity Among MSM, Male-to-Female Transgender Patients, and Female Sex Workers, Lima, Peru, January 2019 to July 2019

Through the exploratory analyses conducted through LASSO logistic regression, we found that among MSM and transgender women the factors correlated with treponemal positivity were reported STI history, education, neighborhood income level, reported condom use, sexual intercourse role, and HIV infection status. Of those, the variables that most strongly predicted treponemal positivity were HIV coinfection compared with lack of HIV coinfection (adjusted odds ratio [aOR], 5.42) and reported STI history compared with no STI history (aOR, 1.54). Higher education compared with high school education only (aOR, 1.53), middle neighborhood income level compared with low income level (aOR, 1.93), lack of reported condom use compared with reported condom use (aOR, 0.60), and receptive sexual intercourse role compared with unknown role (aOR, 1.13) were also associated with treponemal positivity.

### Treatment Data by Syphilis Diagnosis

We also noted that 88% of primary syphilis cases, 100% of secondary syphilis, and 94.3% of unknown latent syphilis were treated with 3 doses of 2.4 million units of benzathine penicillin G (BPG). Of those with a positive RPR result and clinically characterized as having serofast syphilis, 100% were not given treatment (see Table 2).

**TABLE 2:**
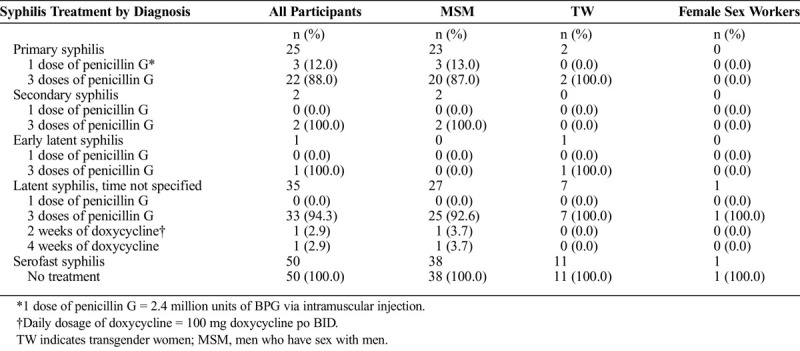
Syphilis Treatment by Diagnosis in High-Risk Patients in Lima, Peru, July 2019

## DISCUSSION

We completed a retrospective chart review of 401 patients at risk for syphilis, 252 MSM, 31 transgender women, and 118 female sex workers, who received a point-of-care rapid treponemal test in Lima, Peru. We found that several factors were associated with treponemal positivity. The majority of patients with reported HIV infection had a positive treponemal test, indicating syphilis infection sometime in their lifetime. Among those that were infected with HIV, frequencies of treponemal positivity were 73.8% among MSM and 55.6% and transgender women. This association is well documented, and several studies have associated recent syphilis with HIV coinfection.^14–16^ Given the high levels of HIV coinfection in this population, we believe that PrEP should be considered as part of the Peru public health response.

Additionally, of those that reported a past STI other than HIV or syphilis, treponemal positivity was also high at 49.0%. In terms of behavioral characteristics, reported sexual practices were associated with varying levels of treponemal positivity. Men who have sex with men who reported insertive anal sex as their primary role had a frequency of treponemal positivity of 26.9%. This figure increased to 47.1% and 45.9% among those who primarily practice receptive anal sex and versatile anal sex, respectively.

Lifetime prevalence of syphilis of MSM (37.7%) and transgender women (54.8%) in our study is higher compared with studies in other countries. One study conducted in Central Brazil in 2015 of MSM and transgender women found lifetime prevalence of syphilis to be 26.3% among MSM and 50% among transgender women.^17^ Another study conducted in 2009 of MSM in Beijing, China found seroprevalence to be 19.8%.^18^ Yet another study of HIV-infected MSM in Turkey in 2020 reported overall seroprevalence to be 28.7%.^19^ Additionally, 1 study of transgender youth in the United States found that 55% of transgender females reported a syphilis diagnosis in their lifetime.^20^ Our findings support high rates of lifetime prevalence of syphilis found in MSM and transgender women around the world. With respect to MSM, this figure is higher than what has been reported in other countries, and among transgender women, our data corroborate a prevalence of ~50% in this particularly at-risk population.

Fewer studies have looked at lifetime prevalence of syphilis in at-risk groups in Peru. One study of high risk, HIV-uninfected MSM and transgender women conducted in a sexual health clinic in Lima, Peru in 2012 reported that 18% of total participants had positive serology for syphilis.^21^ We find in our study that positivity on the treponemal test is 27.6% and 54.5% for MSM and transgender women who are HIV-uninfected, respectively. These numbers are concerning because they may suggest higher lifetime prevalence of syphilis in those key populations in recent years.

In contrast with MSM and transgender women, frequencies of treponemal positivity were low among female sex workers. Of 118 female sex workers, only 4 were positive for treponemal antibodies, a frequency of 3.4%, and notably none of those cases were younger than 30 years. Additionally, among the 4 that were positive for treponemal antibodies, only one had active syphilis infection. Two had nonreactive RPRs, one had an RPR result of 1:1 and was diagnosed clinically as a serofast result, and one had an RPR result of 1:1 and was diagnosed clinically with latent syphilis.

Compared with female sex workers in other countries, the lifetime prevalence of syphilis among the female sex workers in our study (3.4%) is lower than what has been reported in female sex workers in Moscow (13.9%)^22^ and in female prisoners who report having ever received money for sex in Brazil (12.5%).^23^ Our findings were higher than a study of female sex workers in Iran (0.4%).^24^ Notably, none of the female sex workers in our population were infected with HIV while HIV prevalence was reported in Moscow (3.1%)^22^ and Iran (2.1%).^24^ Past studies in Peru corroborate this low incidence of syphilis infection. In a 2002 household based survey of 4485 female sex workers, only 0.8% had an active syphilis infection.^25^ Treponemal positivity may be much lower in female sex workers in Peru compared with other countries due to monthly “control” check-ups that are required to maintain their sex worker license.^26^ These check-ups include a clinical check-up, STI testing, monthly sexual health counseling, and condom distribution. Due to low incidence of syphilis infection in this population, decreasing syphilis testing to once a quarter could potentially save testing resources without impacting the quality of care.

We also found that the clinicians in the STI clinics studied tend to treat recently acquired syphilis with 3 doses of 2.4 million units of BPG via intramuscular injection, which is not in line with the Peruvian national STI treatment guidelines of 1 dose of 2.4 million units of BPG.^27^ This finding indicates potentially wasted medical resources such as the medication itself, as well as additional staff time and patient visits to provide subsequent injections. Additionally, this result may point to a need for stronger educational efforts for clinicians, as the apparent standard of care does not follow national guidelines. It may be that some clinicians treat with 3 doses to approximate prophylaxis from further doses, which could preempt re-infection from partners who do not come in for care. This has been reported to our team as a tactic by physicians in these clinics.

Additionally, we found that 10.1% of the patients of our study population were diagnosed with serofast syphilis and a large percentage of the RPR results of those with treponemal positivity fell between 1:1 and 1:4, dilutions that are not high enough to make a diagnosis of active syphilis infection per national guidelines^27^ (see Fig. 1). Further research is necessary to improve diagnostic strategies for those patients.

Our study has several limitations. First, our study population was not population-based. The medical records used were the clinical records that were accessible to the study team but do not represent all of the patients seen in this period. However, those included are a random selection of clinical histories. Second, we did not repeat treponemal antibody tests, and therefore, there is a chance there were false positives included in the study. We believe this chance is low due to the high specificity of the treponemal antibody test used.^10^ Additionally, the medical records were physical documents, often with incomplete information. These records included standardized questions about sexual behavior over the last 3 months as opposed to lifetime behavior. These data would be most reflective of risk of recently acquired syphilis and does not necessarily reflect lifetime risk. Moreover, it was unknown if the factors listed in the medial record were present at the time of syphilis infection. We also measured income levels indirectly through district of residence, and thus it is possible that it is not reflective of the participant's true socioeconomic status. In addition, transcribing the information to REDCap could have led to potential translational error although we tried to mitigate those effects through training of our data abstractors. Finally, the number of transwomen included in our study was much lower than the number of MSM or female sex workers, giving us limited statistical power to make conclusions about this population. Statistical limitations include using complete case analysis; this reduced the sample size available for analysis and can bias results. Also, utilizing LASSO regression does not provide CIs or *P* values, which help to further quantify significance.

We successfully completed a retrospective chart review of MSM, transgender women, and female sex workers that received a rapid point-of-care treponemal test in Lima, Peru. We found the incidence of lifetime prevalence of syphilis to be high among MSM and transgender women and low among female sex workers. Those results indicate a need for more frequent, regular testing among MSM and transgender women—possibly in conjunction with HIV testing, and appropriate treatment of those shown to be positive. Current testing recommendations for MSM and transgender women include a clinical check-up, STI testing, monthly sexual health counseling, and condom distribution every 3 months. Additionally, given the high frequency of coinfection with HIV and other STIs, current public health strategies should aim to improve syphilis and HIV prevention among those individuals.
